# Blood immune cell profiling in adults with longstanding type 1 diabetes is associated with macrovascular complications

**DOI:** 10.3389/fimmu.2024.1401542

**Published:** 2024-07-01

**Authors:** Xuehui He, Xinhui Wang, Julia van Heck, Bram van Cranenbroek, Esther van Rijssen, Rinke Stienstra, Mihai G. Netea, Irma Joosten, Cees J. Tack, Hans J. P. M. Koenen

**Affiliations:** ^1^ Department of Laboratory Medicine - Medical Immunology, Radboud University Medical Center, Nijmegen, Netherlands; ^2^ Luxembourg Centre for Systems Biomedicine, University of Luxembourg, Belvaux, Luxembourg; ^3^ Department of Internal Medicine, Radboud University Medical Center, Nijmegen, Netherlands; ^4^ Department of Immunology and Metabolism, Life and Medical Sciences Institute, University of Bonn, Bonn, Germany

**Keywords:** type 1 diabetes mellitus, immune cell profile, heterogeneity, multiparameter flow cytometry, machine learning, hierarchical classification

## Abstract

**Aims/hypothesis:**

There is increasing evidence for heterogeneity in type 1 diabetes mellitus (T1D): not only the age of onset and disease progression rate differ, but also the risk of complications varies markedly. Consequently, the presence of different disease endotypes has been suggested. Impaired T and B cell responses have been established in newly diagnosed diabetes patients. We hypothesized that deciphering the immune cell profile in peripheral blood of adults with longstanding T1D may help to understand disease heterogeneity.

**Methods:**

Adult patients with longstanding T1D and healthy controls (HC) were recruited, and their blood immune cell profile was determined using multicolour flow cytometry followed by a machine-learning based elastic-net (EN) classification model. Hierarchical clustering was performed to identify patient-specific immune cell profiles. Results were compared to those obtained in matched healthy control subjects.

**Results:**

Hierarchical clustering analysis of flow cytometry data revealed three immune cell composition-based distinct subgroups of individuals: HCs, T1D-group-A and T1D-group-B. In general, T1D patients, as compared to healthy controls, showed a more active immune profile as demonstrated by a higher percentage and absolute number of neutrophils, monocytes, total B cells and activated CD4+CD25+ T cells, while the abundance of regulatory T cells (Treg) was reduced. Patients belonging to T1D-group-A, as compared to T1D-group-B, revealed a more proinflammatory phenotype characterized by a lower percentage of FOXP3+ Treg, higher proportions of CCR4 expressing CD4 and CD8 T cell subsets, monocyte subsets, a lower Treg/conventional Tcell (Tconv) ratio, an increased proinflammatory cytokine (TNFα, IFNγ) and a decreased anti-inflammatory (IL-10) producing potential. Clinically, patients in T1D-group-A had more frequent diabetes-related macrovascular complications.

**Conclusions:**

Machine-learning based classification of multiparameter flow cytometry data revealed two distinct immunological profiles in adults with longstanding type 1 diabetes; T1D-group-A and T1D-group-B. T1D-group-A is characterized by a stronger pro-inflammatory profile and is associated with a higher rate of diabetes-related (macro)vascular complications.

## 1 Introduction

Type 1 diabetes mellitus (T1D) is a complex disease that involves immunological, environmental and genetic factors in its pathogenesis (1). Studies in non-obese diabetic (NOD) mice have identified the engagement of CD4, CD8 T cells and macrophages in beta-cell destruction (2). Other immune cell types including B cells, NK and NKT cells, as well as dendritic cells (DC) are present in pancreatic islet infiltrates from mice and patients with recent-onset T1D (3–5), indicating that multiple immune cell subsets contribute to the disease development. In children with recently diagnosed T1D, circulating numbers of peripheral NK, DCs and CD8+ effector memory (EM) cells were significantly lower, whereas CD4+ central memory (CM) and CD4+ naïve cells were significantly higher as compared to age-matched healthy controls (6). Next to the involvement of immune cells in T1D pathogenesis and disease progression, immune suppressive regulatory T cells (Treg) are crucial in the prevention/delay of disease progression. Lack of Treg accelerated T1D disease onset in a murine model (7). Treg of T1D patients showed an impaired capacity to control effector T cells, and the patients CD4+ cells were thought to be relatively resistant to regulation by Treg (8, 9).

Heterogeneity of T1D exists in epidemiology, genetics, number of autoantibodies, islet infiltrating lymphocytes, and rate of disease progression. Obviously, multiple pathways are involved in T1D occurrence and progression, and various pathogenetic mechanisms result in a similar disease outcome i.e. beta-cell autoimmunity. Given this multifactorial disease induction, the concept of T1D endotypes has been proposed in order to reflect T1D disease subtypes (10–13). T1D endotype-1 includes patients diagnosed at young age (< 7-years old) and characterized with early beta cell destruction and aggressive insulitis with abundant infiltration of CD8+ T cells and CD20+ B cells, whereas T1D endotype-2 includes patients diagnosed in adolescence or adulthood that have fewer infiltrating CD20+ B cells (13, 14). Once T1D disease is established, patients have a high risk to develop vascular complications (15). There is ample evidence that the immune system is also involved in the development of diabetic-associated vascular complications. For example, pro-inflammatory biomarkers are positively associated with cardiovascular risk (16, 17).

In contrast to studies on the immune system of paediatric diabetes patients, there is limited information available regarding the immune system in longstanding type 1 diabetes. Here we applied multiparameter flow cytometry combined with a machine learning based clustering analysis to identify peripheral immune cell profile heterogeneity in adults with longstanding T1D.

## 2 Methods

### 2.1 Study cohort

Western-European individuals with type 1 diabetes (T1D, n = 242, age = 51.8 ± 16.3 years) were selected from the outpatient diabetes clinic of the Radboud university medical centre, Nijmegen, the Netherlands as previously described (17). Age/sex/BMI-matched healthy individuals (HC, n = 37, age = 53.0 ± 10.5 years) were carefully selected from the baseline data (before vaccination) of a cohort of healthy individuals vaccinated with BCG (http://www.humanfunctionalgenomics.org). Inclusion criteria were a diagnosis of type 1 diabetes (based on clinical criteria with or without anti-GAD positivity) and age over 18 years. Participants were excluded when pregnant, used antibiotics up to 4 weeks before inclusion, or were rescheduled when experiencing a fever in the week before inclusion. Information about disease history and complications was collected from the corresponding medical records. The average duration of diabetes was 28.5 ± 15.7 years, and the mean onset-age of diabetes was at 23.2 ± 13.5 years. Detailed demographic information including age, sex, BMI, and clinically defined diabetes-related complications are listed in **Table 1**. The study was approved by the ethical committee of Radboud University Nijmegen (NL-number: 54214.091.15). Experiments were conducted according to the principles expressed in the Declaration of Helsinki. All study participants provided written informed consent.

**Table 1:** Demographic and clinical parameters of T1D and HC cohort.

	HC (n = 37)	T1D			
		In total (n = 242)	T1D-group-A (n = 50^#^)	T1D-group-B (n = 191^#^)	p-values (group-A vs -B)
Age (years)	53 ± 10	52 ± 16	54 ± 16	52 ± 16	0.55
Male sex (%)	50%	54%	66%	51%	0.07
BMI (Kg/m^2^)	24.4 ± 3.6	25.9 ± 4.3 * (5 missing values)	26.4 ± 4.3* (2 missing values)	25.7 ± 4.4* (3 missing values)	0.29
Disease duration (years)	—	29 ± 16* (2 missing values)	28 ± 16	29 ± 15* (2 missing values)	0.77
Onset-age of T1D(years)	—	23 ± 14* (2 missing values)	25 ± 16	23 ± 13* (2 missing values)	0.63
Onset-age <= 7years	—	10%* (2 missing values)	12%	10%* (2 missing values)	0.61
Insulin usage (unit/kg)	—	0.66 ± 0.45* (7 missing values)	0.70 ± 0.48* (2 missing values)	0.65 ± 0.43* (5 missing values)	0.59
HbA_1c_ level (mmol/mol)	—	64 ± 15* (1 missing value)	63 ± 13	64 ± 15* (1 missing value)	0.91
hsCRP (µg/ml)	—	1.8 ± 2.5	2.2 ± 3.6	1.7 ± 2.1	0.38
***Macrovascular compl.***	—	17%* (2 missing values)	**35%**	**14%** * (2 missing values)	**0.02**
Coronary arterial disease	—	10%* (2 missing values)	16%	8%* (2 missing values)	0.12
Peripheral arterial disease	—	7%* (2 missing values)	10%	6%* (2 missing values)	0.39
Stroke	—	6%* (2 missing values)	12%	4%* (2 missing values)	0.06
***Microvascular compl.***	—	73%* (20 missing values)	78%* (5 missing values)	72%* (15 missing values)	**0.45**
Nephropathy	—	18%* (4 missing values)	24%	17%* (4 missing values)	0.29
Neuropathy	—	47%* (5 missing values)	52%	47%* (5 missing values)	0.54
Retinopathy	—	64%* (28 missing values)	65%* (7 missing values)	64%* (21 missing values)	0.95

1. Demographic characteristics were shown as mean ± S.D.

2. Patients with macro-/micro-vascular complications were defined as ‘yes’’ when at least one of the three-listed complications present.

3. ^#^One T1D patient was classified neither to T1D-group-A nor T1D-group-B. Thus, it is skipped during the detailed comparison between patient subgroups.

4. ^*^Missing values exist for the corresponding variables.

5. BMI, body mass index; hsCRP, high-sensitivity C-reactive protein; compl., complications.

### 2.2 Blood processing

Venous blood was sampled in sterile 10mL ethylenediaminetetraacetic acid (EDTA) tubes (Vacutainer system, Becton Dickinson) from all participants that presented non-fasting between 8.00 and 11.00 am at the outpatient clinic. Peripheral blood was processed within 1–4 hours after collection. This was similar for the healthy control cohort.

Fresh peripheral blood cells were counted using a Coulter Ac-T Diff^®^ cell counter (Beckman Coulter, Brea, USA) that was calibrated daily. Erythrocytes were lysed by incubating whole blood with the lysis buffer containing 3.0 M NH4Cl, 0.2 M KHCO3 and 2 mM Na4EDTA for 10 minutes on the bench. The remaining leukocytes were washed twice with 25 ml phosphate-buffered saline 1x (PBS, Braun, Melsungen, Germany) and centrifuged at 452g for 5 min at room temperature. Before performing the flow cytometry staining, cells were resuspended in 200 µl of PBS enriched with 0.2% bovine serum albumin (BSA, Sigma-Aldrich, Zwijndrecht, Netherlands). Isolation of PBMCs was performed by density centrifugation of EDTA anticoagulated blood diluted 1:1 in pyrogen-free PBS over Ficoll-Paque (VWR, Amsterdam, The Netherlands) as described previously (18).

### 2.3 Flow cytometry

Multicolour flow cytometry was used to study the innate and adaptive immune cell composition in peripheral blood. In brief, cells (5.0 x 10^6^ cells/mL) were stained with five distinct and complementary 10-color monoclonal antibody panels (**Supplementary Table S1**): 1. General panel; 2. T cell panel; 3. B cell panel; 4. intracellular T cell/Treg panel; 5. chemokine receptor (CCR) panel. Stained samples were measured on a Navios flow cytometer (Beckman Coulter, Fullerton, CA, USA), equipped with three solid-state lasers (488 nm, 638 nm, and 405 nm). Flow cytometry data were analysed using Kaluza software version 2.1. The gating strategies applied for the B cell panel and chemokine receptor panel are listed in **Supplementary Figure S1**. Gating strategies of other panels were previously described (18). **Supplementary Tables S1**, **S2** showed the detailed FACS staining panels and the parent population of cell subsets. The absolute number of white blood cells (WBC) per ml of blood determined by the cell counter was used to calculate the absolute numbers of CD45+ WBC cell subsets as measured by flow cytometry (18).

### 2.4 *In vitro* cytokine production capacity

*In vitro* functional analysis of cytokine production by PBMCs was performed as described previously (17). 5×10^5^ PBMCs were stimulated with pathogens Staphylococcus aureus (1×10^6^/mL) or heat-inactivated Candida albicans hyphae (1×10^6^/mL) for 24 hours (TNFα, IL-10) or 7 days (IFNγ and IL-17). Culture supernatants were collected at the end of the incubation period and stored at −20°C until cytokines were measured using enzyme-linked immunosorbent assay. The production of TNFα, IL-10, IFNγ and IL-17 cytokines was measured using commercial ELISA kits (R&D Duoset ELISA Systems) according to the manufacturer’s instructions. The assay detection ranges are; 15.6 - 1000 pg/mL (TNFa), 31.2 - 2000 pg/mL(IL-10), 9.4 - 600 pg/mL (IFNγ) and 15.6 - 1000 pg/mL (IL-17).

### 2.5 Statistical analyses

R-Studio (R Foundation for Statistical Computing, Vienna, Austria) with R version 4.1.3 statistical software was used for the statistical analyses. A two-side p value less than 0.05 was considered significant, with multiplicity correction using the Benjamini-Hochberg false discovery rate correction. Comparison of numeric variables was performed using Wilcoxon signed-rank test, and comparison of categorical variables using Pearson’s Chi-Squared test. Individual’s age and/or sex were adjusted when necessary. Spearman correlation of immune cell subsets was calculated using Hmisc package in R, and only one of the strongly correlated cell subsets (rho >0.9) was included in further analysis (see details in **Figure S2**). Principal component analysis (PCA) was performed using FactMineR package. The top two principal component (PC1 and PC2) were used to project samples in a bi-dimensional scatter plot. One-way ANOVA was used to test the difference of PCs between cohorts.

### 2.6 Elastic-net classification model

A multivariate model was applied to determine which cell subsets were important to stratify patients from healthy controls. Since the sample size of this study is relatively small in relation to the number of immune cell subsets identified (in total 112 subsets), and due to the potential collinearity of various immune cell subsets, a penalized logistic linear regression approach, i.e. the elastic-net (EN) classification model was applied by using the h2o package in R. The elastic net regression achieves a balance between the lasso and ridge regularization which could mitigate both multicollinearity and model overfitting while simultaneously selecting the important variables out of a large set of explanatory predictors (19). The input variables of the model are the percentages of immune cell subsets generated by the above-described flow cytometry analysis and individual’s age and sex. The response variable is the individual’s status, i.e. either T1D vs HC or T1D-group-A vs T1D-group-B. The grid search of hyperparameter alpha was set as from 0 to 1 with a 0.1 interval. The model generation applied the iteratively reweighted least squares method to find an optimal lambda value. The EN model was constructed based on the training-dataset (70% of total dataset) with five-fold cross-validation and verified with the test-dataset (the other 30% of total dataset). Standardized coefficient magnitudes ranging from 0 to 1 were used to report the scaled variables importance. The most explanatory variables were selected based on the sum of the relative influence of variables achieving 90% (see details in **Supplementary Table S3**). Hierarchical clustering of the most explanatory cell subsets was performed using the ward.D2 method. Please note one healthy individual was assigned to T1D-group-A and one T1D patient was classified to the healthy-subgroup. Thus, this specific patient was omitted in further comparative analysis of patient subgroups.

## 3 Results

### 3.1 T1D patients and healthy controls reveal distinct circulating immune cell profiles

**Figure 1:**
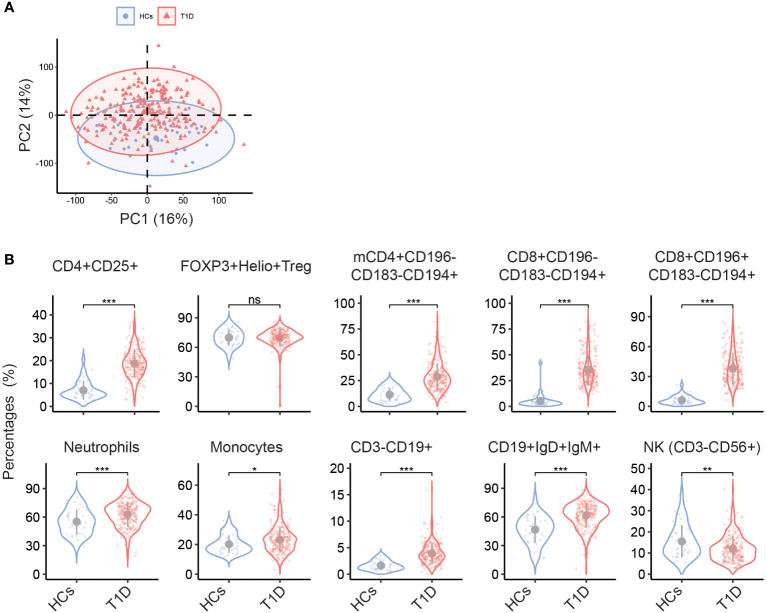
Different circulating immune cell subsets in T1D and HC. **(A)** Biplot of the first two principal components (PCs) of percentages of immune cell subsets determined by multiparameter flow cytometry. **(B)** Violin plots show the univariate comparison of cell percentages between T1D (n = 242) and HC (n = 37), adjusted for the covariates age and sex. Each dot represents one individual. Grey dot indicates the mean value, and grey vertical line represents the standard deviation. *, p<0.05; **, p<0.01; ***, p<0.001; ns, not significant. mCD4, memory CD4+ cells.

Demographic characteristics of T1D and healthy controls (HC) are listed in **Table 1**. HC and T1D patients were matched for age, BMI and sex. Principle component analysis (PCA) of immune cell subset percentages as obtained by flow cytometry revealed a significant separation of T1D and HC especially on PC2 which explained 14% of total variance (p<0.05, **Figure 1A**).

Univariate analysis revealed multiple immune cell subsets that were significantly different between T1D and HC (details are listed in **Supplementary Table S2**). As compared to HC, a clear increase in CD4+CD25+ T cells was noticed in T1D (**Figure 1B**). Since CD25 is not only a T cell activation marker but also highly expressed on regulatory T cells (Treg), we further gated on Treg cells that showed high expression of CD25 but low expression of CD127 within all CD4+ cells and used FOXP3 and Helios to track changes in Treg. We found no difference in percentage of FOXP3+Helios+ Treg in T1D vs HC (**Figure 1B**), indicating that the observed increase of CD4+CD25+ T cells was mainly confined to activated T subsets. Other T cell subsets, especially those expressing the chemokine receptor CCR4/CD194, including memory CD4+CD196-CD183-CD194+, CD8+CD196-CD183-CD194+, and CD8+CD196+CD183-CD194+ were significantly higher in T1D (both percentages and absolute numbers, **Figure 1B** and **Supplementary Figure S3**). In the T1D cohort also increased proportions of neutrophils, monocytes, total CD3-CD19+ B cells and CD19+IgD+IgM+ subsets were observed, while NK cells were decreased.

**Figure 2:**
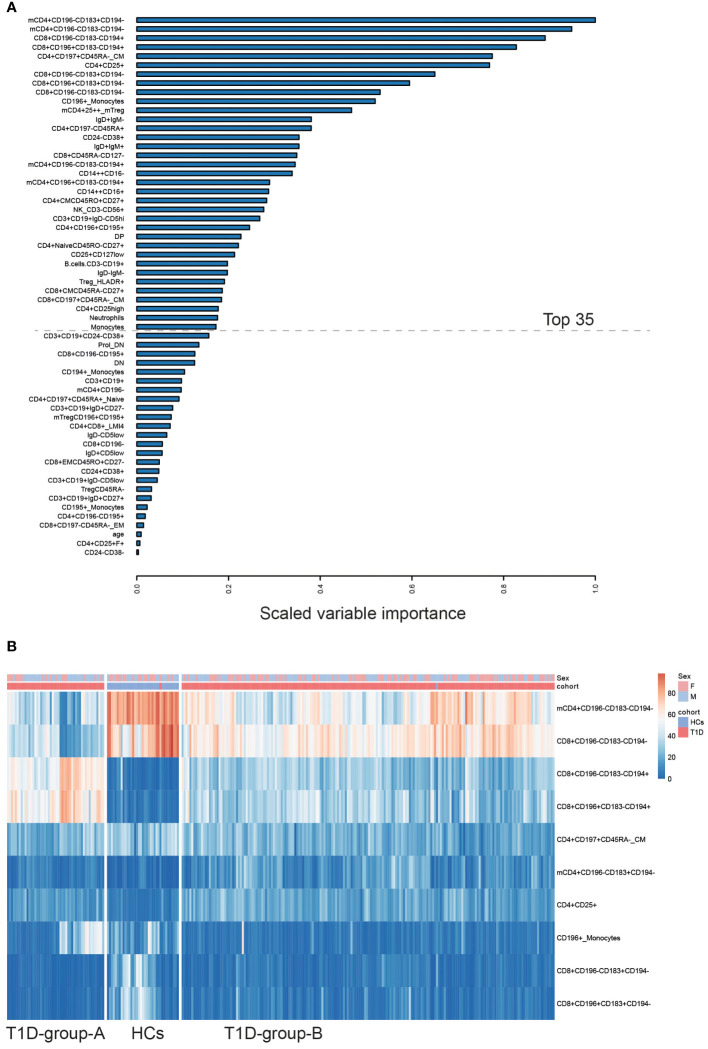
Elastic net based ranking of circulating immune cell subsets and hierarchical clustering of T1D patients and healthy controls. The response variable of the EN model was the individual’s cohort status, i.e. HC vs T1D. The input variables were the percentages of immune cell subsets plus individual’s age and sex (see details in the *Method* section). The scaled variable importance value was used to rank the weight of each explanatory variables. **(A, top)** Variable importance plot showed all explanatory variables selected by the EN-model. The most explanatory immune cell subsets contributing to 90% of the classification (top-35) are those above the dashed line. **(B, bottom)** Hierarchical clustering using the top-35 most explanatory immune cell subsets revealed three clusters; one healthy control cluster and two distinct T1D-patient subgroups. The example heatmap shows the top-10 of immune cell subsets contributing to the classification. Cohort status and sex of every individual are listed on the top of heatmap. T1D (n = 242), HC (n = 37). F: female; M: male; mCD4, memory CD4+ cells.

To select and rank the most discriminating peripheral immune cell subsets that distinguish T1D from HC, we applied a penalized linear regression model i.e. elastic net (EN) model which performs feature selections while creating the binary classification model. As shown in **Figure 2A**, 59 out of 112 immune cell subsets plus individual’s age were selected by the EN model. The scaled variable importance quantitatively measures how on average a variable contributes to the classification model. Age showed only a minor contribution to the classification of T1D vs HC since its scaled variable importance was just above zero, whereas the top 35 of the selected immune cell subsets contributed to 90% of the classification (see details in the **Supplementary Table S3**). Thus, we named such top-35 immune cell subsets selected by the EN model as the most explanatory variables.

Consistent with previous reports showing that T cells are major drivers of T1D disease progression, the majority of immune cells selected by the model (**Figure 2A** and **Supplementary Figure S3B**) were CD4 and CD8 T cell subsets displaying distinct expression of CD25, CD45RA, CCR7/CD197 or CD27, and which in addition express one or multiple chemokine receptors (CXCR3/CD183, CCR4/CD194, CCR6/CD196). Together, this indicates that T cell activation, differentiation and migration capabilities within the T cell compartment are modified in T1D. Next to T cells, various B cell subsets, monocytes expressing CCR6/CD196, neutrophils, and NK cells were also selected by the model (**Figure 2A**), suggesting that multiple immune cell types contribute to distinguish T1D patients from healthy individuals. Notably, hierarchical clustering using the most explanatory variables (i.e. the top-35 immune cell subsets) clearly revealed three subgroups; two T1D-patient subgroups and one healthy control group. **Figure 2B** lists the example of the most explanatory cell subsets. All healthy controls except one individual were clustered together and were separated from T1D patients. Interestingly, all T1D patients except one were clustered into two separate subgroups, which we refer to as T1D-group-A and T1D-group-B. The patient (#DM229) that was classified to the HC cluster was omitted in subsequent patient-subgroups analysis.

### 3.2 Two T1D patient subgroups are revealed by their blood immune cell profile

**Figure 3:**
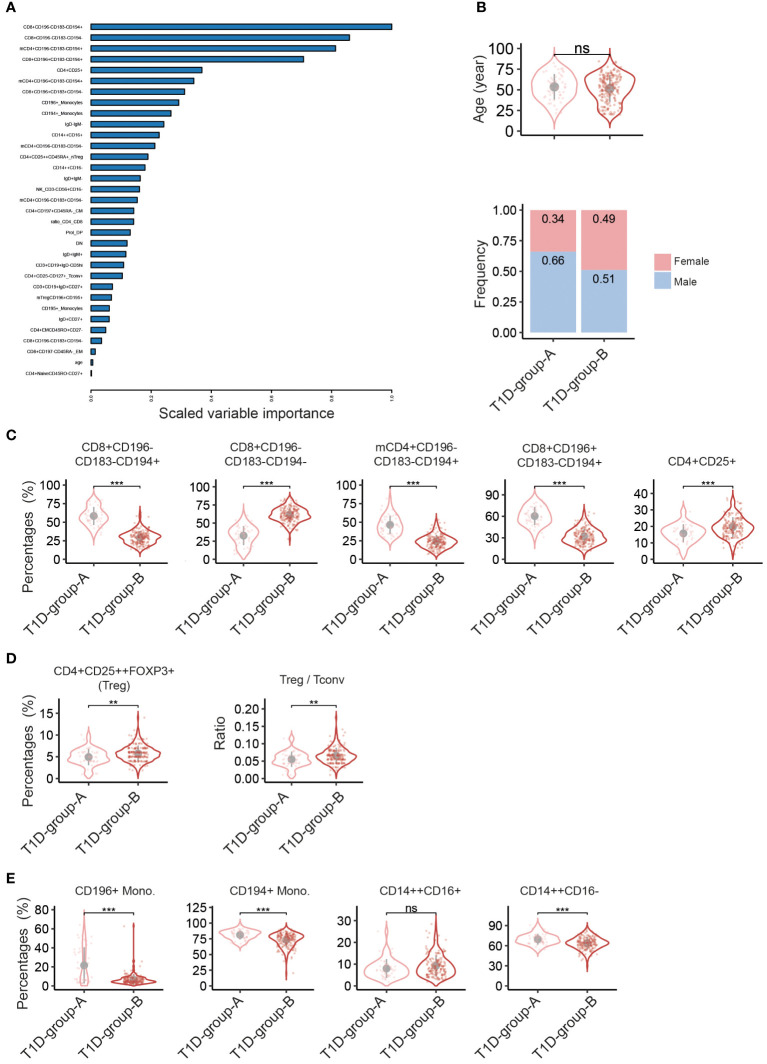
Discriminating peripheral immune cell profiles classify two T1D- patient groups. Elastic net (EN) modelling of T1D patients only. The response variable of the EN model was patient’s subgroups, i.e. T1D-group-A vs T1D-group-B. The input variables were the percentages of immune cell subsets plus patient’s age and sex. **(A)** Variable importance plot shows all variables selected by the EN-model (33 out of 114 input explanatory variables, Y-axis). **(B**-**E)** Violin plots showing univariate comparison, adjusted for the covariates age and sex, between T1D-group-Avs T1D-group-B patients for **(B)** age (top) and sex (bottom), **(C)** top-5 explanatory immune cell subsets as selected by the EN model, **(D)** percentages of FOXP3+ Treg and ratio of CD4+ regulatory T cells: CD4+ conventional T cells (Treg/Tconv) based on absolute cell counts of CD4+CD25++Foxp3+ and CD4+CD25- cells, and **(E)** percentages of monocyte subsets. T1D-group-A (n = 50), T1D-group-B (n = 191). Grey dot indicates the mean value, and grey vertical line represents the standard deviation. **, p<0.01; ***, p<0.001.; ns, not significant. mCD4, memory CD4+ cells. F, Female; M, Male.

To study in detail the difference of the immune cell profiles within the T1D subgroups, a new EN-model was constructed focusing on the patient flow cytometry data only and excluding the HC data. In total thirty-three explanatory variables, mainly immune cell populations, were selected by the model (**Figure 3A** and **Supplementary Figure S4A**), including various T cell subsets, B subsets, NK cells and monocytes. Age, albeit with a very low scaled variable importance, was also selected by the model, but no significant difference was reached based on the direct univariate comparison (**Figure 3B**, top). The differences in the immunological profile between T1D-group-A vs T1D-group-B were neither driven by onset-age of diagnosis, duration of disease, BMI, or glycated haemoglobin (HbA1c) (**Table 1**). Slightly more male patients were present in T1D-group-A than in T1D-group-B (66% vs 51%, X^2^_Pearson(1)_ = 3.39, p = 0.0656, **Figure 3B**, bottom).

**Figure 3C** shows the top-5 immune cell subsets selected by the model; percentages of CD8+CD196-CD183-CD194+, memory CD4+CD196-CD183-CD194+ and CD8+CD196+CD183-CD194+ were significantly higher in T1D-group-A, whereas percentages of CD8+CD196-CD183-CD194- and CD4+CD25+ were lower as compared to T1D-group-B. Analysis of absolute numbers of cell subsets showed a similar results (**Supplementary Figure S4C**). Interestingly, the percentage of FOXP3+ Treg and the ratio of Treg: conventional T cells (Treg/Tconv) were significantly lower in T1D-group-A than in T1D-group-B (**Figure 3D**), suggesting that the immune system of T1D-group-A is less tailored to immune tolerance. Furthermore, CD196 or CD194 expressing monocytes and CD14++CD16- classical monocytes were also enriched in T1D-group-A (**Figure 3E**). Several B cell subsets as well as CD4 or CD8 effector memory cells were also selected by the EN-model, but univariate comparison did not reach the statistical significance (**Supplementary Figure S4B**).

### 3.3 T1D-group-A shows increased pro-inflammatory cytokine production and exclusive cytokine/immune cell subset correlations

**Figure 4:**
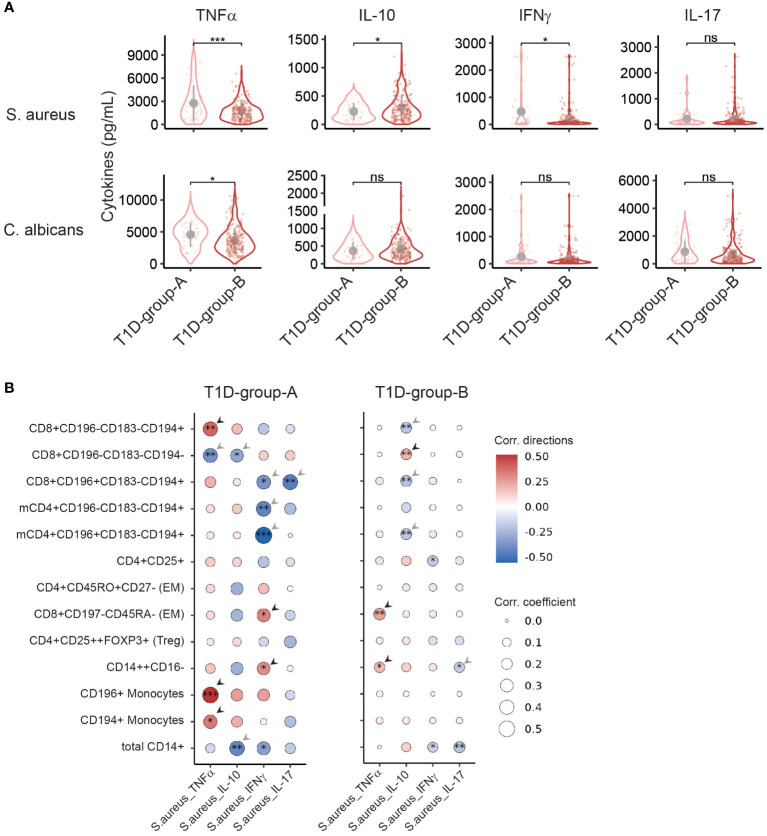
T1D-group-A shows increased pro-inflammatory cytokine production and exclusive cytokine / immune cell subset correlations. Cytokines production as measured by ELISA of PBMCs stimulated *in vitro* with *S. aureus* (top panels) or *C*. *albicans* (bottom panels). Innate cytokines TNFα and IL-10 were measured 24 hours after stimulation and adaptive cytokines IFNγ and IL-17 were measured 7 days after stimulation. **(A)** Violin plots show the cytokines production in T1D-group-A vs T1D-group-B. Age and sex covariates were adjusted. **(B)** Pearson’s correlation of the percentages of cell subsets and cytokines produced upon stimulation with *S.aureus* in T1D-group-A (left panel) or T1D-group-B (right panel). Dot size represents the absolute correlation coefficients and the colour scale indicates the correlation direction. Arrows indicate the mutual exclusive correlation patterns in T1D-group-A (n = 50) vs T1D-group-B (n = 191). Asterisk(s) within the dot indicate the significance of correlation p-values. *, p<0.05; **, p<0.01; ***, p<0.001; ns, not significant. memCD4, memory CD4+ cells; Mono., monocytes; EM: effector memory.

To determine potential changes of immune cell functionality in T1D-group-A and -B, PBMCs were stimulated with the pathogens Staphylococcus aureus or Candida albicans and cytokines released in cell culture supernatant were measured by ELISA. Upon stimulation with S. aureus, higher amounts of the pro-inflammatory cytokines TNFα and IFNγ but a lower amount of anti-inflammatory cytokine IL-10 were found in T1D-group-A as compared to T1D-group-B (**Figure 4A**). No difference was observed regarding the production of IL-17. Stimulation with C. albicans also led to higher TNFα production in T1D-group-A. These results indicates that next to differences in the immune cell profile, also functional differences exist between T1D-subgroups.

Next, the association of cytokine production following S. aureus stimulation and the proportion (cell percentages) of most explanatory immune cell subsets in both patient subgroups was evaluated. Exclusive correlation patterns were observed between the T1D subgroups (**Figure 4B**; **Supplementary Figure S5**). Regarding correlations with TNFα, for T1D-group-A positive correlations with CD8+CD196-CD183-CD194+ T cells and CD194+ and CD196+ monocytes were found, while in T1D-group-B CD8+CD197-CD45RA- EM T cells and CD14++CD16- monocytes correlated positively to TNFα. Furthermore, for T1D-group-A CD8+CD196-CD183-CD194- T cells revealed a negative correlation with TNFα (**Figure 4B**). With respect to correlations with IL-10, for T1D-group-A negative correlations with CD8+CD196-CD183-CD194- T cells and total CD14+ monocytes were found. For T1D-group-B negative correlations were found with CD8+CD196-CD183-CD194+, CD8+CD196+CD183-CD194+ and CD4+CD196+CD183-CD194+, while CD8+CD196+CD183-CD194- correlated positively to IL-10 (**Figure 4B**). With respect to correlations with IFNγ, only clear exclusive correlations in T1D-group-A were observed with CD4 and CD8 T cell subsets (**Figure 4B**). Finally, regarding IL-17, for T1D-group-A a negative correlation with CD8+CD196+CD183-CD194+ T cells was found, and for T1D-group-B CD14++CD16- classical monocytes showed a negative correlation (**Figure 4B**). The differential immune cell - cytokine production correlations of T1D-group-A and T1D-group-B further support the presence of these patient subgroups within type-1 diabetes.

### 3.4 Higher prevalence of macrovascular disease in T1D-group-A

**Figure 5:**
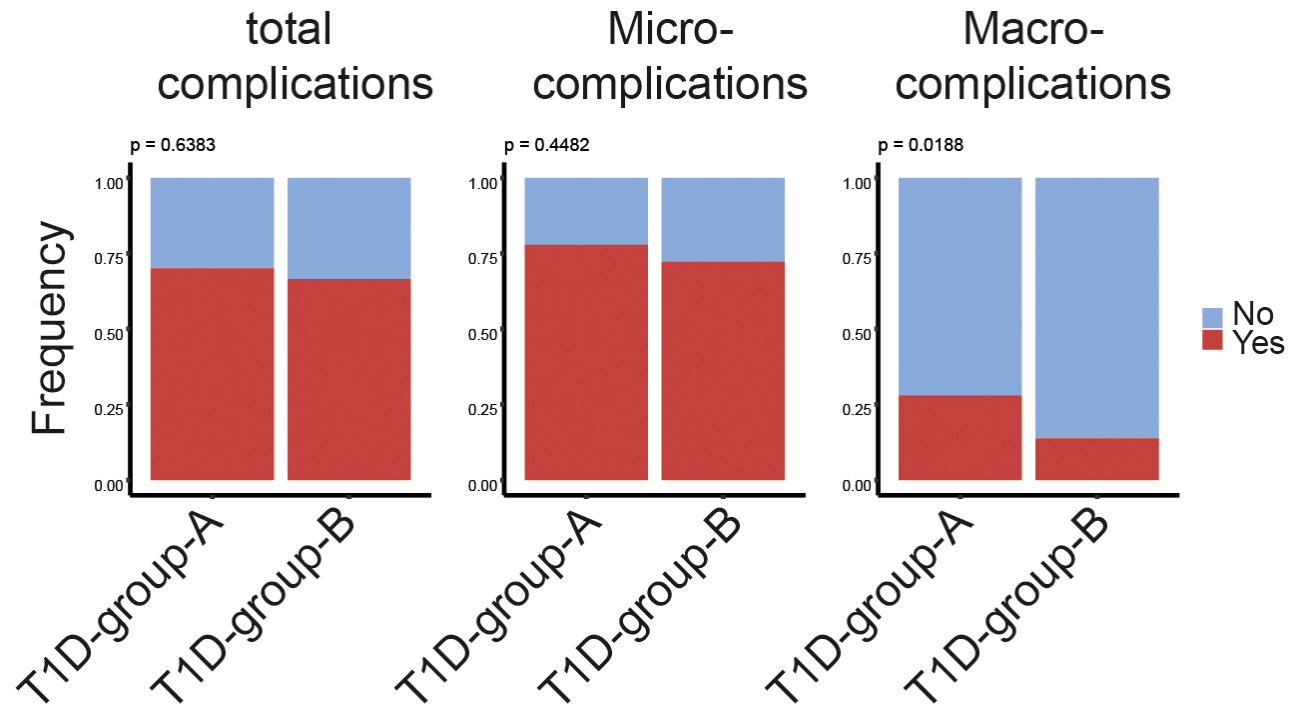
Higher prevalence of macrovascular disease in T1D-group-A. Bar plots showing the percentage of patients associating with or without diabetes-related complications regardless of macro-/micro-complications (left panel), only macrovascular complications (middle panel) or only microvascular complications (right panel)in T1D-group-A (n = 50) versus T1D-group-B (n = 191). The p-value of the Chi-squared test is listed on the top of the bars. Yes indicating patient associating with at least one of the corresponding complications listed on the top of the bars.

Having characterized the above described T1D subgroups, we next compared the prevalence of diabetes-related vascular complications including three microvascular (diabetic nephropathy, neuropathy, and retinopathy) and three macrovascular complications (stoke, coronary arterial disease, and peripheral arterial disease). There was no observed significant difference between patients subgroups when comparing the frequency of patients with or without any of the six complications (**Figure 5**, left panel). Interestingly, when we further compared the appearance of either only macro- or micro-complications, a significantly higher prevalence of macrovascular complications was observed in T1D-group-A (**Figure 5**, middle panel), while there was no clear difference for the presence of microvascular complications (**Figure 5**, right panel).

## 4 Discussion

Emerging evidence suggests the existence of disease endotypes within type 1 diabetes based on early childhood age disease onset (10, 11, 20, 21). By analysing the flow cytometry based immune cell profiling data using a machine-learning approach, we here show that adults with longstanding T1D can be partitioned into two immunologic subgroups characterized by a different circulating immune cell profile. Patients belonging to T1D-group-A, as compared to T1D-group-B, revealed a more proinflammatory phenotype characterized by a lower percentage of FOXP3+ Treg, higher proportions of CCR4 expressing CD4 and CD8 T cell subsets, a lower Treg/Tconv ratio and an increased proinflammatory cytokine (TNFα/IFNγ) producing potential upon stimulation, while the anti-inflammatory IL-10 producing capacity was reduced. Clinically, patients in T1D-group-A had more frequent macrovascular complications.

We and others have shown that blood immune cell profiling approaches are a powerful tool for detailed characterization of immune cell subsets in peripheral blood of healthy individuals and patients (18, 22–24). The association of peripheral immune cell changes and disease progression of type 1 diabetes has been shown in several studies, especially in newly diagnosed diabetes patients (6, 7, 23–26). Here, focusing on adults with longstanding T1D, we show that they have a more active immunological profile as compared to age-matched healthy controls. Consistent with previous studies in children with established diabetes showing an increase of circulating neutrophils and a decrease of NK cells (7, 24), we also observed enhanced neutrophil and diminished NK proportions in adults affected for more than 20-years with diabetes. However, in contrast to the lower circulating monocyte proportions found in paediatric patients diagnosed for less than 2-years with diabetes (27), we observed enhanced percentages and absolute numbers of monocytes in adults with longstanding T1D as compared to healthy controls. Within our T1D cohort, classical monocytes CD14+CD16-, CD196+ monocytes and CD194+ monocytes were significantly higher in T1D-group-A than in T1D-group-B, indicating that heterogeneity in circulating monocytes with specific migratory capacities and possibly function can develop during the progression of diabetes.

Increases in B cells, CD4 and CD8 T cells numbers have been reported in paediatric as well as adult patients at onset of T1D (23, 24). Our current data in adults with longstanding diabetes, as compared to healthy controls, demonstrates that B cell numbers remain increased, more specifically naive IgD+IgM+ B cells were increased. Alterations of B cells and their association with islet dysfunction have been reported (28, 29). It is known that marginal zone (MZ) B cells circulate in human blood as IgM+IgD+CD27+ B cells (30) and the expansion of the MZB cell compartment has been observed in type 1 diabetes murine model (31). However, we do not find differences in IgM+IgD+CD27+ B cells in either T1D vs HC, or in T1D-group-A vs T1D-group-B (data not shown).

In contrast to the B cell numbers, total CD4 or CD8 T cell proportions in adults with longstanding T1D are similar to healthy controls. However, we noticed a clear increase of CCR4/CD194 expressing T subsets in our patient cohort. Especially, two CD8 subsets (CD8+CD196-CD183-CD194+ and CD8+CD196+CD183-CD194+) seem to be crucial to distinguish T1D patients from healthy controls as well as T1D-group-A versus T1D-group-B, since they were ranked as the top-5 most relevant cell subsets selected by the EN-model (see **Figures 2A**, **3A**). CCR4 is a chemokine receptor for thymus and activation regulated chemokine (TARC) and macrophage-derived chemokine (MDC). Human CCR4+CD8+ T cells were shown able to produce the cytokines TNFα and IL-4 (32). Although, the diabetes-causing potential of CCR4-expressing T cells has been highlighted in an NOD mice model (33), another study suggests that recruitment CCR4-bearing Treg to the pancreatic islets may actually prevent murine autoimmune diabetes (34). Additional studies are needed to ascertain the precise relevance of CCR4 on specific immune cell subsets in the pathogenesis of type 1diabetes.

Systematic comparison of the immune cell profile among patients with longstanding T1D in the current study revealed two distinct immune phenotypic subgroups; T1D-group-A and T1D-group-B. An established T1D endotype is associated to early life disease onset in patients that are diagnosed < 7-years of age that is characterized by aggressive insulitis with abundant CD8+ T and CD20+ B cell infiltration (12, 13), The percentage of patients with onset-age of diagnosis in our cohort under 7-years was only slightly higher in T1D-group-A (12%) than in T1D-group-B (10%) and thus seems not to explain the immune phenotypic subgroups as we present here. Serum glucose concentrations and glycaemic control also seem to influence the circulating immune cells phenotype and function. For example, tolerogenic DCs derived from patients under suboptimal glycaemic control (HbA1C > 58 mmol/mol) have reduced tolerogenic capabilities compared to those from patients under optimal control (HbA1c < 58 mmol/mol] (35). However, in general our patients had suboptimal glycaemic control (mean HbA1c is 64 mmol/mol), and no statistical difference between T1D-group-A and in T1D-group-B was observed. Instead, the prevalence of diabetes related macrovascular complications as well as the total numbers of observed macro-/micro-complications were higher in T1D-group-A than T1D-group-B. Given the cross-sectional design of our study, our data cannot differentiate between cause and consequence in the relationship between atherosclerosis and the T1D-group-A specific immune cell profile. Currently, the clinical relevance of classifying patient subgroups as identified in our study seems limited to a potential increased risk of developing macrovascular complications. Further work is required to confirm the current findings.

The strength of current study is that we have applied a comprehensive immunological phenotyping in a large group of rather homogeneous patients with longstanding type 1 diabetes. Due to the complexity of the immune system itself and (potential) multicollinearity among immune cell subsets, a multivariate model that analyses more than two variables and their relationship as we demonstrate here represents an alternative unbiased tool to identify disease subgroups. To our knowledge, immune cell profiling information in adults with longstanding T1D remains limited. Only Apostolopoulou et al. reported a positive association of lower percentage of CD8 with fasting glycemia level in adults diagnosed for 5-years with T1D (36). Teniente-Serra et al. performed a comparison of immune cell changes among healthy controls, adults at onset of T1D and those with established disease (23). Our observations prompt further investigation of both soluble and cellular inflammatory mediators and their relationship with the occurrence of diabetes related cardiovascular disease.

Some limitations of this study should be underlined, including the cross-sectional design, data on pathogens exposure in our T1D cohorts is lacking, and the fact that the presence of complications was based on questionnaires, which may be less reliable. Additionally, only the circulating immune cell compositions with potential biological variation were investigated. We envisage that inclusion of additional data and larger study populations will further refine disease classification and defining T1D endotypes by for example additional inclusion of more clustering variables such as information on autoantibodies, HLA genotypes and circulating cytokine profiles.

In conclusion, analysis of multiparameter flow cytometry data using the combination of an elastic-net regression model and hierarchical clustering revealed two distinct immunological subgroups in adults with longstanding type 1 diabetes. T1D-group-A is featured by a more pro-inflammatory phenotype, characterized by a lower percentage of Treg, higher percentages of effector T cell subsets expressing CCR4 and an increased production of pro-inflammatory cytokines. Monitoring of the immune cell profile might help to identify diabetes endotypes, which could be relevant in the context of longstanding complications and optimized diabetic care.

## Data availability statement

The raw data supporting the conclusions of this article will be made available by the authors, without undue reservation.

## Ethics statement

The study was approved by the ethical committee of Radboud University Nijmegen (NL-number: 54214.091.15). The studies were conducted in accordance with the local legislation and institutional requirements. The participants provided their written informed consent to participate in this study.

## Author contributions

XH: Visualization, Validation, Supervision, Software, Resources, Project administration, Funding acquisition, Writing – review & editing, Writing – original draft, Methodology, Investigation, Formal analysis, Data curation, Conceptualization. XW: Visualization, Data curation, Conceptualization, Formal analysis, Writing – review & editing, Writing – original draft, Methodology. Jv: Resources, Writing – review & editing, Data curation. Bv: Formal analysis, Writing – review & editing. Ev: Formal analysis, Writing – review & editing. RS: Conceptualization, Writing – review & editing. MN: Writing – review & editing, Supervision, Conceptualization. IJ: Writing – review & editing, Supervision, Conceptualization. CT: Conceptualization, Funding acquisition, Supervision, Visualization, Writing – review & editing. HK: Conceptualization, Data curation, Formal analysis, Funding acquisition, Investigation, Methodology, Project administration, Resources, Software, Supervision, Writing – review & editing, Writing – original draft, Visualization, Validation.
