# Supplementary figures and images for: Blood immune cell profiling in adults with longstanding type 1 diabetes is associated with macrovascular complications
Source: Front Immunol. 2024 Jul 1;15:1401542. doi: 10.3389/fimmu.2024.1401542 (PMC11246869; doi:10.3389/fimmu.2024.1401542)

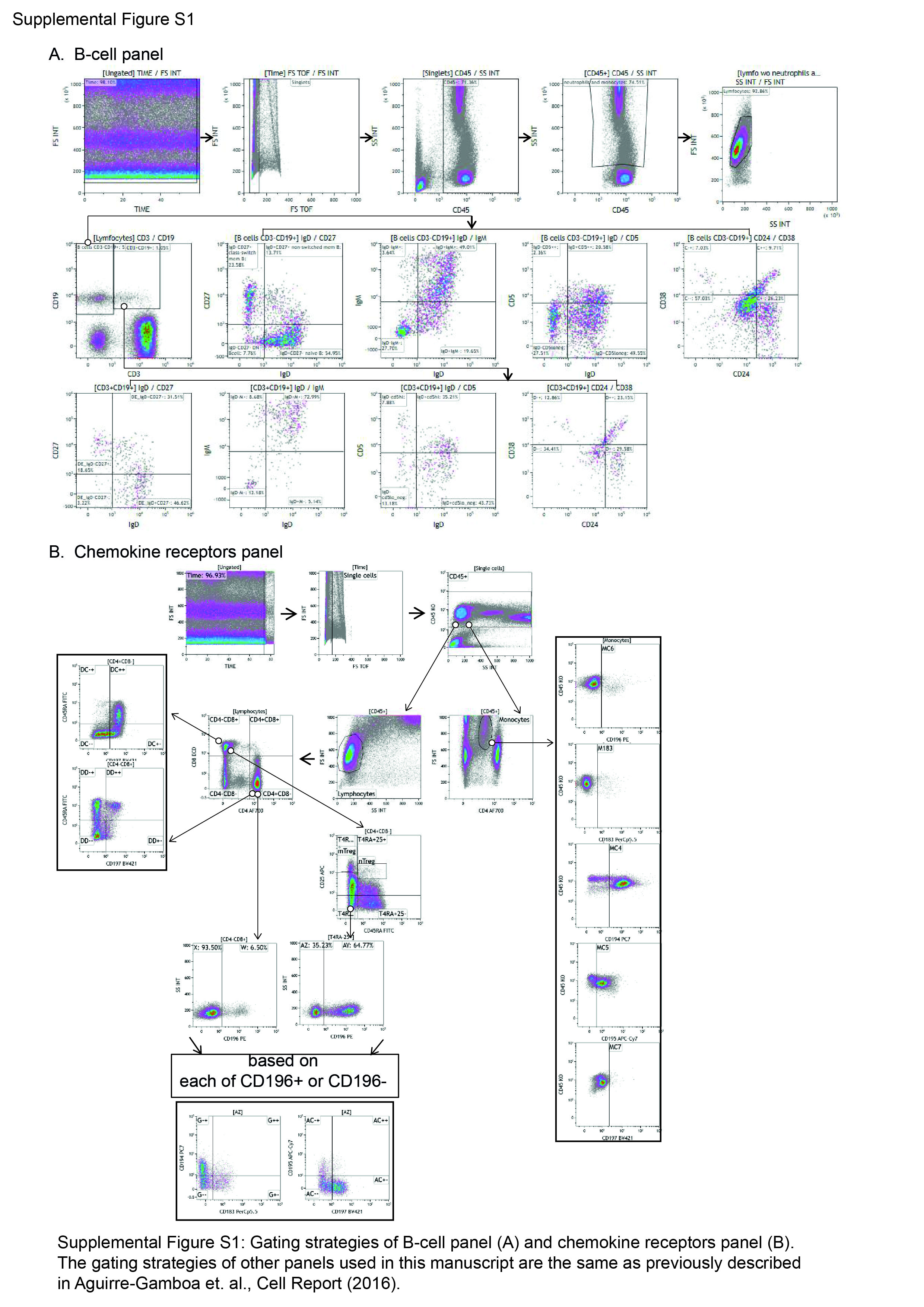

Supplement: Supplementary file 1 [file Image_1.jpeg]

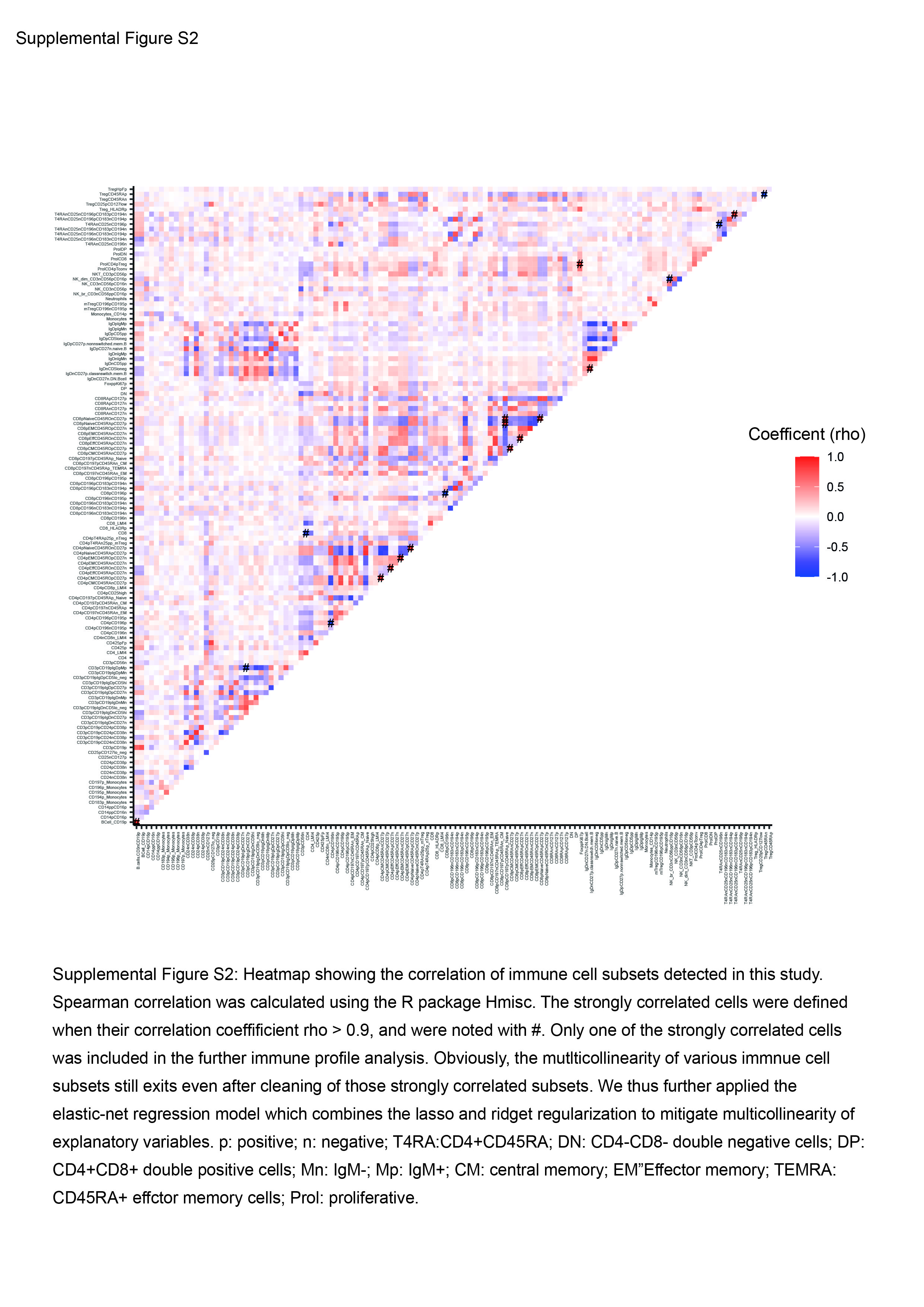

Supplement: Supplementary file 2 [file Image_2.jpeg]

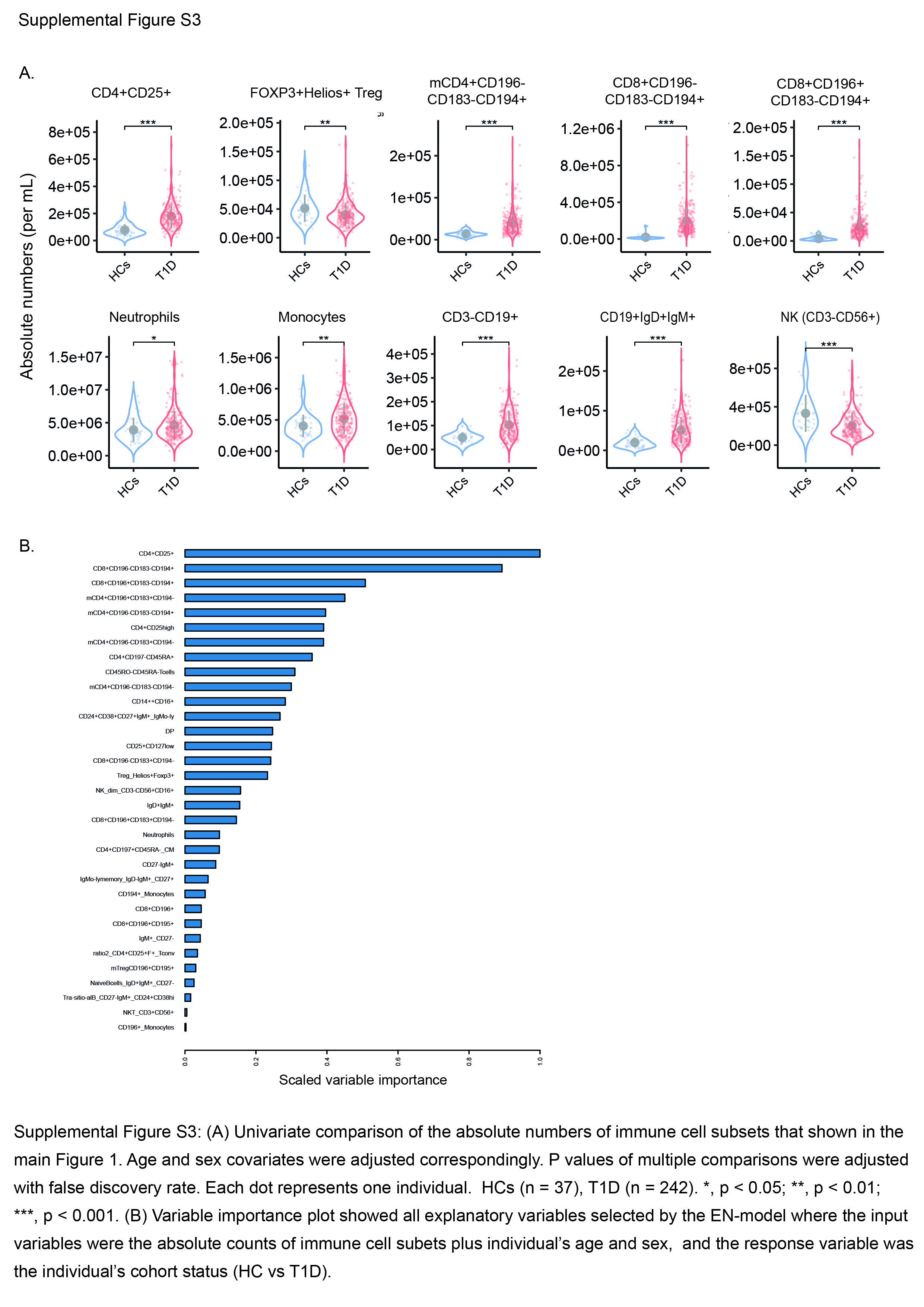

Supplement: Supplementary file 3 [file Image_3.jpeg]

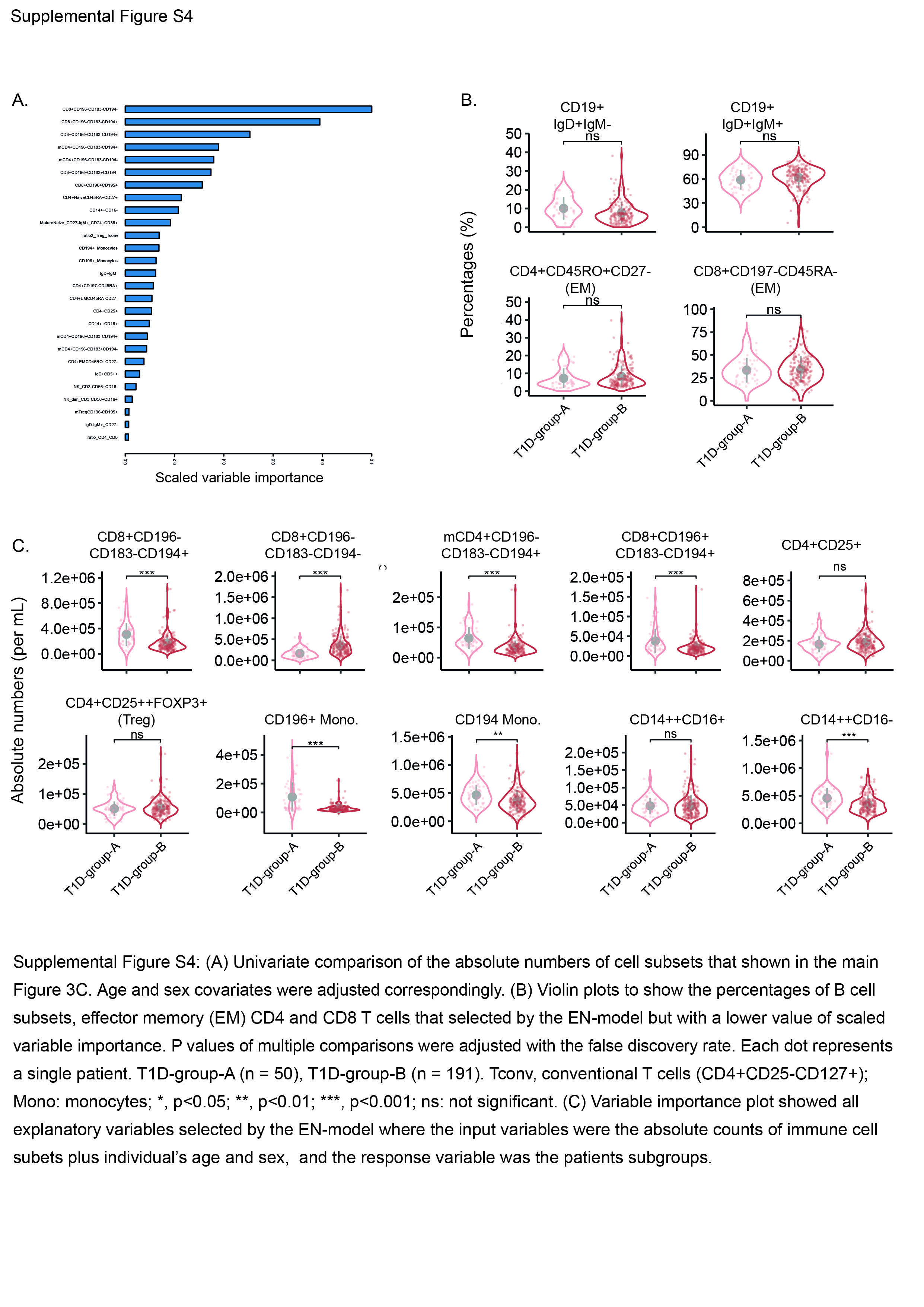

Supplement: Supplementary file 4 [file Image_4.jpeg]

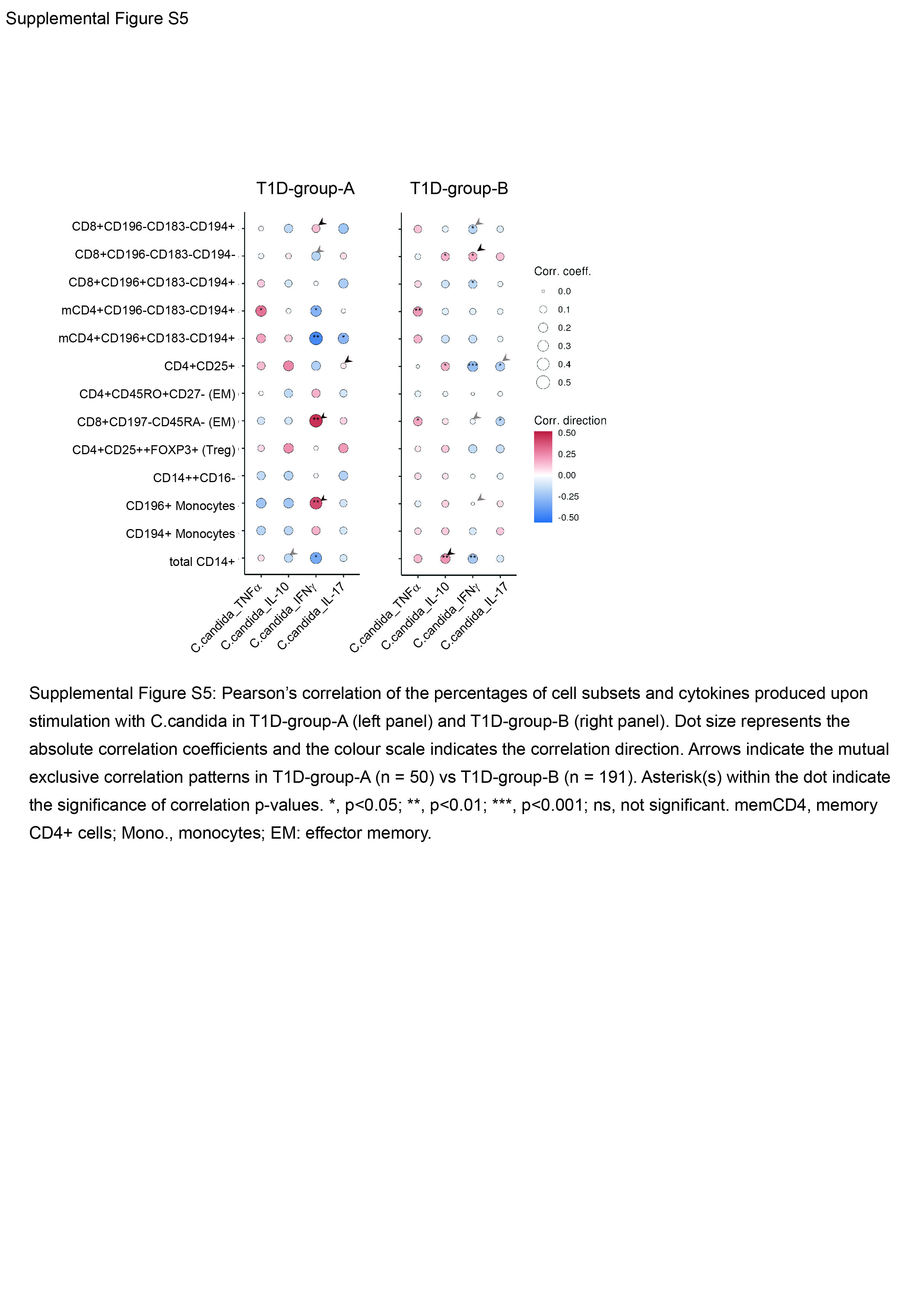

Supplement: Supplementary file 5 [file Image_5.jpeg]
